# Repellent, Irritant and Toxic Effects of 20 Plant Extracts on Adults of the Malaria Vector *Anopheles gambiae* Mosquito

**DOI:** 10.1371/journal.pone.0082103

**Published:** 2013-12-23

**Authors:** Emilie Deletre, Thibaud Martin, Pascal Campagne, Denis Bourguet, Andy Cadin, Chantal Menut, Romain Bonafos, Fabrice Chandre

**Affiliations:** 1 UR Hortsys, Cirad, Montpellier, France; 2 Plant Health Department, ICIPE, Nairobi, Kenya; 3 UMR CBGP, INRA-CIRAD-IRD-Montpellier SupAgro, Montpellier, France; 4 Institut des Biomolécules Max Mousseron, Faculté de Pharmacie, Montpellier, France; 5 Montpellier SupAgro, USAE, Montpellier, France; 6 UMR MiVEGEC, IRD-CNRS-UM1-UM2, Montpellier, France; University of Tours, France

## Abstract

Pyrethroid insecticides induce an excito-repellent effect that reduces contact between humans and mosquitoes. Insecticide use is expected to lower the risk of pathogen transmission, particularly when impregnated on long-lasting treated bednets. When applied at low doses, pyrethroids have a toxic effect, however the development of pyrethroid resistance in several mosquito species may jeopardize these beneficial effects. The need to find additional compounds, either to kill disease-carrying mosquitoes or to prevent mosquito contact with humans, therefore arises. In laboratory conditions, the effects (i.e., repellent, irritant and toxic) of 20 plant extracts, mainly essential oils, were assessed on adults of *Anopheles gambiae,* a primary vector of malaria. Their effects were compared to those of DEET and permethrin, used as positive controls. Most plant extracts had irritant, repellent and/or toxic effects on *An. gambiae* adults. The most promising extracts, i.e. those combining the three types of effects, were from *Cymbopogon winterianus, Cinnamomum zeylanicum* and *Thymus vulgaris.* The irritant, repellent and toxic effects occurred apparently independently of each other, and the behavioural response of adult *An. gambiae* was significantly influenced by the concentration of the plant extracts. Mechanisms underlying repellency might, therefore, differ from those underlying irritancy and toxicity. The utility of the efficient plant extracts for vector control as an alternative to pyrethroids may thus be envisaged.

## Introduction

*Anopheles gambiae* (Giles, 1902) complex are major vectors responsible for the transmission of *Plasmodium* spp., particularly *Plasmodium falciparum*, which is the most hazardous protozoan parasite causing malaria infection in humans [1]. In 2010, approximately 3.3 billion people were exposed to malaria resulting in 655,000 deaths [2]. Although it still remains one of the most severe human diseases across the world, the overall incidence of malaria has fallen by 17% between 2010 and 2011. This decrease has been ascribed to an enormous progress in the control of malaria due to the use of efficient tools, such as rapid diagnostic tests in combination with treatments like artemisinin-based combination therapy (ACT) against *P. falciparum,* and control with indoor residual spraying or long-lasting insecticide-treated mosquito nets. These strategies have contributed to improved public health in many countries [3]. Nevertheless, vector management is under the threat of resistance development to pyrethroids. Indeed, resistance to pyrethroids has been reported in 27 countries in sub-Saharan Africa, underscoring the urgent need to find other alternatives to these insecticides [2], [4]. Historically, the search for novel compounds to be used in vector control has focused on their lethal effects [5]. Nevertheless, other effects such as repellency or irritancy [6] may be used to reduce vector–host contact. Pyrethroids have four main effects on mosquitoes causing: (i) a spatial repellent effect, i.e. deterrence of adults from entering treated rooms; (ii) a contact irritant effect, i.e. short-lived settling of mosquitoes on treated bednets or walls; (iii) an anti-feedant effect, i.e. blood feeding inhibition of female mosquitoes and 4) toxic effect, i.e. a knock down and mortality effect [7].

According to Mathews & Mathews [8], a compound can be considered a spatial repellent, when its odour causes a shifting of animals away from the source. Spatial repellency has increasingly been given attention over the last few years since it has the potential to reduce the encounters between hosts and vectors [5]. A compound is considered irritant whenever insects move away after contact with it [9]. Compounds like pyrethroids or DDT increased insect activity because of their irritant effect [10].

Plants contain compounds such as repellents, anti-feedants, and growth regulators preventing attack from phytophagous insects, but some of these compounds are also repellent for haematophagous insects [11]. This could be an evolutionary relict from plant-feeding ancestors since many plant compounds evolved as repellents to phytophagous insects [12]. Plants are used worldwide to protect people from haematophagous arthropods and numerous studies report repellent effects of essential oils [11], [13], [14], [15], [16]. These natural compounds are biodegradable, environmentally friendly and popular [17], and they generally have a low mammalian toxicity [18]. Moreover, traditional medicine is largely plant-based (herbs or shrubs) and is available at low cost in most tropical areas [14].

Essential oils present several interesting properties. First, they easily penetrate insect cuticle, which increases their bioavailability [16]. This property could be of interest if it resulted in shortened stay of insects on treated surfaces. Second, essential oil compounds such as acyclic or monocyclic monoterpenes are small and volatile molecules that might have spatial repellency properties. For example, insect sensilla are specialized for detecting odorants and have been shown to respond to volatile monoterpenes [16]. Finally, active compounds in essential oils may have specific mode of action, which makes them good alternatives to the use of pyrethroids.

Large screening programmes of chemicals traditionally used for vector control have aimed to generate baseline data for comparison with novel compounds. Using a high-throughput screening system (HITSS), compounds can be rapidly assayed and their effect on mosquito behaviour explored [19]. This study aimed at identifying the most promising plant extract(s) to complement the existing collection of molecules used in the control of malaria vectors. The broad aim of our study was to adapt the HITSS, originally developed for *Aedes aegypti,* [6], [19], [20], [21] to perform assays on *An. gambiae*, to: 1) assess any spatial repellent, contact irritant and/or toxic effects of 20 plant extracts, 2) determine whether the influence of these extracts is concentration-dependent and 3) assess the potential of the selected candidates by comparing their effects with those induced by pyrethroid or neurotoxic insecticides. Among the 20 plant extracts, we identified three that could be used to augment the existing methods of malaria vector control.

## Materials and Methods

### Mosquitoes

Behavioural assays were performed on female *An. gambiae* originating from the insecticide susceptible reference strain “Kisumu”. This strain, originally collected in Kenya in 1953, has been reared at LIN-IRD, Montpellier, France. The insecticide susceptibility of the Kisumu strain was confirmed with World Health Organization (WHO) diagnostic doses (i.e. 4% DDT, 0.75% permethrin) and is controlled every 4 months as recommended by the iso 9001 norm. The colony has been maintained in a climatic room at 27±2°C, 80±10% RH and with a photoperiod cycle of 12 h Light: 12 h Dark. Mosquito larvae were fed with fish food. Emerged adults were placed in 25×25×25 cm cages and fed with 10% honey solution. Females used in the bioassays were from batches of non-blood-fed mosquitoes (4 to 7 days after emergence). Each test was performed three times on 20 females. This was because previous experiments to determine a suitable sample size required for statistical power showed that three replications of 20 females was the smallest number of replicates with the best accuracy for visual observation and with the lowest manipulating time.

### Products

A list of plant was established from the literature [9], [11], [13], [14], [15], [16], [22] based on the major compounds of plant extracts, plant extract effects on insects, and their non-toxicity to humans. The 20 plant extracts were selected among this list of plant for their effects on insect with a very different chemical composition described in literature and confirmed by the provider and composed by one or two major compounds (Table 1). This choice should permit the relation between the chemical composition and the behavioural response. DEET (Sigma Aldrich, France; CAS: 134-62-3) and permethrin (Sigma Aldrich, France; CAS 52645-53-1) were used as positive controls.

**Table 1:** Plant extracts chosen from the literature [9], [13], [14], [16], [21], [53] for their effects on insects, non-toxicity to humans and main compounds.

Common name	Scientific name	Extract form,extracted organ	Major compounds (%)^1^	Provider
Aframomum	*Aframomum pruinosum*	Essential oil, leaf	*E*-(R)-nerolidol (95%)	IBMM*, France
Cinnamon	*Cinnamomum zeylanicum*	Essential oil, bark	Cinnamaldehyde (80%)	Nactis, France
Citronella	*Cymbopogon winterianus*	Essential oil, leaf	citronellal (34%) – geraniol (22%) –citronellol (12%)	Nactis, France(Lot 4001850)
Coleus	*Plectranthus tenuicaulis*	Essential oil, leaf	Epoxyocimene (74.4%)	IBMM, France
Coriander	*Coriandrum sativum*	Essential oil, seed	(+)-linalool (72%)	Fabster, France
Cumin	*Cuminum cyminum*	Essential oil, seed	Cuminaldehyde (30%)	Ipra, France(Lot 902560)
Dill	*Anethum graveolens*	Essential oil, seed	(+)-carvone (60%) – limonene (30%)	IBMM, France
Eucalyptus	*Eucalyptus globulus*	Essential oil, leaf	1,8-cineole (81%)	Huiles & Sens, France(Lot B38037)
Geranium	*Pelargonium graveolens*	Essential oil, leaf	citronellol (41%) – geraniol (18%)	IBMM, France
Ginger	*Zingiber officinalis*	Essential oil, root	Zingiberene (30%)	Ipra, France(Lot 902724)
Lemon	*Citrus limon*	Essential oil, fruit	Limonene (95%)	Capua, Italy(Lot 20500)
Lemongrass	*Cymbopogon citratus*	Essential oil, leaf	Citral (geranial, neral) (75%)	IBMM, France
Litsea	*Litsea cubeba*	Essential oil, leaf	Geranial (45%), neral (32%)	IBMM, France
Pennyroyal	*Mentha pulegium*	Essential oil, leaf	(+)-pulegone (87%)	IBMM, France
Neem	*Melia azadirachta*	Vegetal oil, seed	azadirachtin (<1%)	Huiles & Sens, France(Lot 00028/11)
Pepper	*Piper nigrum*	Essential oil, seed	ß-caryophyllene (30%), limonene (14%),pinenes (14%)	IBMM, France
Rosemary	*Rosmarinus officinalis*	Biologic hydrolat, leaf	1,8-cineole (<1%), camphene (<1%),camphor (<1%)	Huiles & Sens, France(Lot EB815N002)
Savory	*Satureja montana*	Essential oil, leaf	Carvacrol (47%), γ-terpinene (18%),p-cymene (13%)	Huiles & Sens, France(Lot B854002)
Solidage	*Solidago canadensis*	Essential oil, leaf	Germacrene D (32%) - Limonene (13%)	Huiles & Sens, France (Lot A2)
Thyme	*Thymus vulgaris L.*	Essential oil, leaf	Thymol (35%), p-cymene (23%),carvacrol (15%)	Huiles & Sens, France (Lot A2)

1 The percentage composition of the essential oil xas computed by the normalization method from GC/FID analyses, response factors being taken as one for all compounds.

* Institut des Biomolécules Max Mousseron, Montpellier, France.

For each product (the 20 plant extracts, DEET [N,N-diethyl-3-méthylbenzamide] and permethrin), solutions were prepared at 0.01, 0.1 and 1% (volume/volume) diluted in a solvent constituted by 1/3 ethanol and 2/3 silicone oil in Dow Corning® 556 fluid. These three concentrations were chosen after preliminary assays and based on published data [6].

All papers used during the day were treated the morning at the same time. In spatial repellency assays, 3.3 mL of a same solution was deposited at 1.5 cm from the edge of 13×30 cm chromatography papers. Treated papers were allowed to dry at room temperature and used 1 hour later. Papers of the same size were also treated with 3.3 mL of solvent and later used in control assays. One paper is used for three replicates. For contact irritancy and toxicity assays, 2 mL of a same solution was deposited on 12×15 cm chromatography papers. Papers of same size were also treated with 2 mL of solvent, and solvent for the control assays. After drying at room temperature for 30 min, treated papers were stored at 4°C and used 2 to 4 hours later, the time to do the spatial repellency bioassays. Different papers were used in each replicate. For each plant extract, DEET, and permethrin, solutions at 0.01, 0.1 and 1% corresponded to 0.001, 0.01 and 0.1 µl of product per cm^2^, respectively. For DEET (permethrin), dilutions of 0.01, 0.1 and 1% corresponded to 0.55 (0.34), 5.5 (3.4) and 55 (34) nmoles of active ingredient per cm^2^ (a.i./cm^2^).

### Bioassays

Bioassays were conducted between 10 am and 6 pm at 24±1°C and 50±10% RH. For each product, each concentration was replicated three times, i.e. three replicates per concentration in the three types of assays: spatial repellency, contact irritancy and toxicity. For each product, all assays were performed the same day. For each type of assay, the control was first evaluated (three replicates) then the lowest concentration was evaluated (three replicates), followed by the mid-level concentration (three replicates), then the highest concentration (three replicates). The HITSS was washed at the end of each testing day and only one plant extract was tested per day. This protocol reduced the risk of contamination and interactions between volatile compounds. The HITSS was cleaned overnight in the TFD4 detergent (Franklab S.A., France) at 20% for the parts that were in contact with the treated paper (see below) and at 10% for any other parts. The material was rinsed and allowed to dry before reuse. To reduce the risk of contamination, a plastic clear film (Laser transparency films, Apli®, Spain) was placed between the treated chamber (see below) and the treated paper. A new film was used for each test.

#### a) Spatial repellency assays

**Figure 1:**
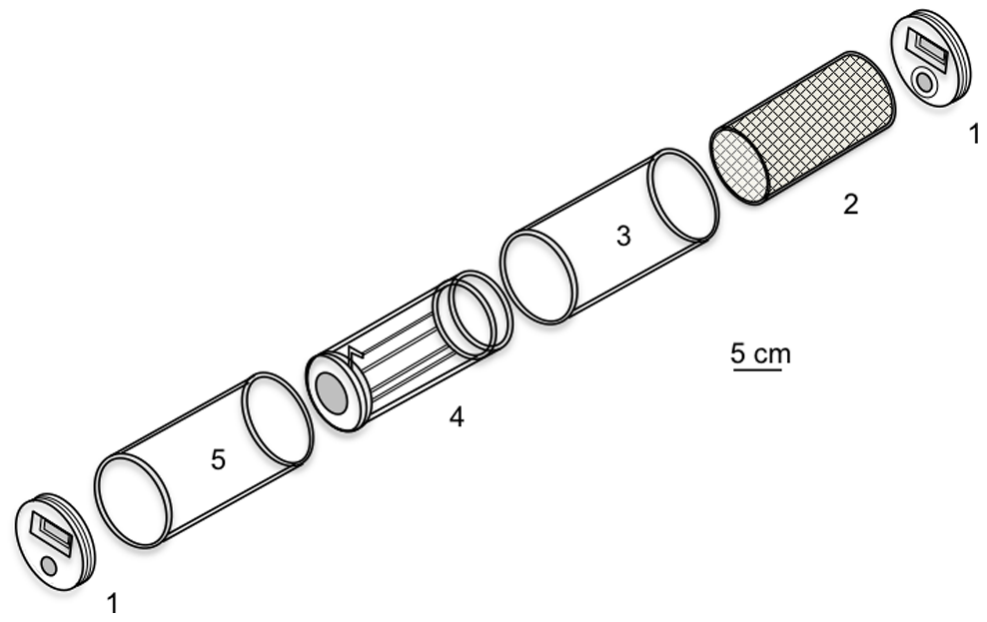
Schematic drawing of a modified HITSS system, used to test spatial repellency. The spatial repellency assay components are: 1, end cap; 2, metallic net; 3, treated chamber; 4, linking section (with a butterfly valve); 5, untreated chamber (adapted from Grieco et al. [18]).

The HITSS, originally developed for *Ae. aegypti* [6], [19], [20], [21], was adapted for *An. gambiae.* The original HITSS is composed of three chambers in a row. The two extreme chambers correspond to the treated and untreated chambers, respectively. *Ae. Aegypti* are introduced in the third chamber, located in the middle of the HITSS [19]. During the experiment mosquitoes have the choice to stay in this middle chamber or to move, either in the treated or in the untreated chamber. Grieco *et al*. [19] used this choice test and considered a spatial activity measure. However, this choice test was not adequate in *An. gambiae* since this species exhibits much lower activity than *Aedes* spp. Furthermore, *An. gambiae* is not clearly attracted or repelled by light or by any external warm source. Hence, irrespective of the experimental condition, *An. gambiae* mosquitoes stayed in the middle chamber of the original HITSS. Consequently, the HITSS used in our experiments (Figure 1) had only two chambers, the treated (part #3) and untreated (part #5) chambers. Treated papers, with products or with only the solvent (for controls), were rolled around the inner surface of the treated chamber and maintained by means of part #4. The inner surface of the untreated chamber (part #5) was covered by a chromatograph paper, which was treated with neither product nor solvent. Thus the two chambers, treated and untreated, received an equivalent brightness. A metallic net (part #2) of 0.3 µm mesh was inserted within part #4, preventing direct mosquito contact with the treated paper. Two end caps (part #1) covered both sides of the HITSS. Part #4 contained a ‘butterfly’ valve that allowed mosquitoes to freely move between the untreated and treated chambers. During assays, the HITSS was held steady and parallel to the bench top by a cradle of 1.3-cm-thick Plexiglas made by Plexi d’Oc, St Gely du Fesc, France.

For each assay, 20 mosquitoes were transferred into the treated chamber using mechanical aspiration. After a 30-sec acclimation period, the butterfly valve was opened for 10 minutes. Mosquitoes moving from the treated chamber to the untreated chamber were referred as ‘escaped’. Conversely, mosquitoes remaining in the treated chamber were referred as ‘stayed’. At the end of the test, the butterfly valve was closed and the number of ‘escaped’ and ‘stayed’ mosquitoes recorded. Before running a new replicate, mosquitoes were removed from the system using CO_2_ anaesthesia and the HITSS system partially disassembled in 5 minutes (chambers were disconnected and the end caps opened) to drive off any volatilized compounds. The assays for a given product were considered as valid whenever less than 20% of ‘escaped’ mosquitoes were in the control replicate. Spatial activity index used by Grieco *et al.* [19] for *Ae. aegypti* was not realistic for *An. gambiae* because the HITSS used in our experiments did not allow adult mosquitoes to make a choice. Thus, we decided to estimate the ability of a plant extract to repel mosquitoes by the proportion of ‘escaped’ mosquitoes: the higher the proportion of escaped, the stronger the spatial repellency effect.

#### b) Contact irritancy assays

**Figure 2:**
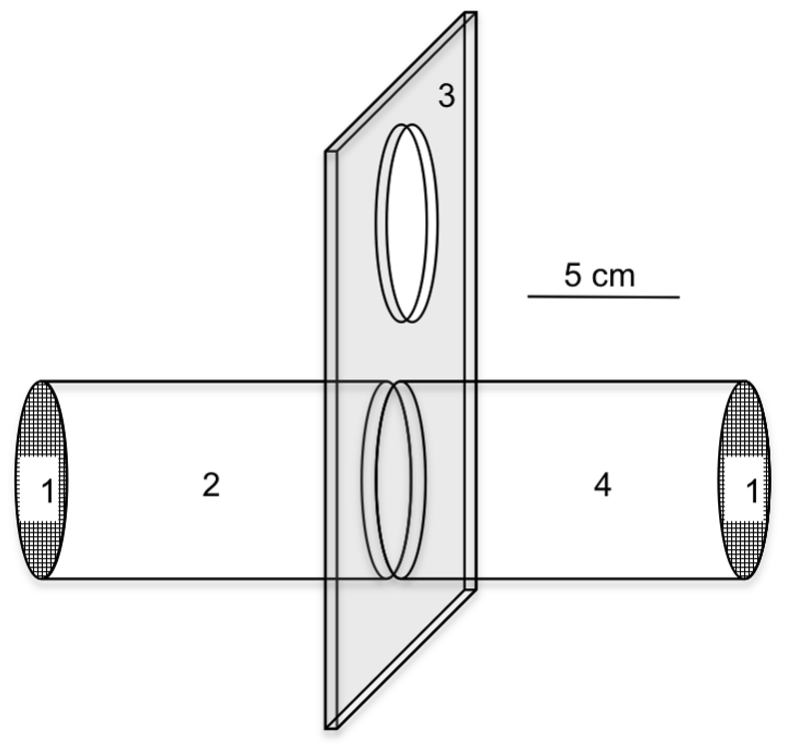
Schematic drawing of a simplified WHO diagnostic test kit for measuring insecticide susceptibility/resistance status in adult malaria mosquitoes, used to demonstrate contact irritancy. The contact irritancy assay components are: 1, end cap covered by net; 2, treated chamber; 3, linking section (guillotine valve); 4, untreated chamber (adapted from Grieco et al. [18]).

These assays were performed with the tube used in the WHO test kit (Figure 2). A treated paper, with the diluted product or with solvent only (for controls) was put in the ‘treated’ tube and an untreated paper (i.e. a paper treated with neither a product nor solvent) in the ‘untreated’ tube. Twenty mosquitoes were initially placed inside the treated tube through the small hole of the slide unit (part #3). The untreated tube was fixed in the opposite part of the apparatus. Then, after a 30-sec acclimation period, the slide unit was opened for 10 minutes allowing the mosquitoes to freely move from tube to tube. Mosquitoes moving from the treated tube to the untreated tube were considered as ‘escaped’. Conversely, mosquitoes staying in the treated tube were referred as ‘stayed’ mosquitoes. Once the guillotine valve was closed, the number of ‘escaped’ and ‘stayed’ mosquitoes in each tube was recorded. For each product, the assays were considered valid whenever the proportion of ‘escaped’ mosquitoes in the control assay (the assay performed with a paper treated with only the solvent) was lower than 50%. In case this ratio was >50%, all replicates were re-run until the ratio was <50% in the control assay. The contact irritant activity of a product was estimated based on the proportion of ‘escaped’ mosquitoes, a high activity translating into high proportions.

#### c) Toxicity assays

Toxicity assays were performed using a WHO test kit [23]. Twenty mosquitoes were exposed during 1 hour to a treated paper (with products or with the solvent only) in the treated tube used for the contact irritancy assay. Mosquitoes were then transferred into an untreated tube with 10% sucrose solution and maintained at 27°C and 80% RH. The number of dead and alive *An. gambiae* was recorded after 24 hours. The assay was considered valid whenever there were less than 10% of dead mosquitoes in the control (treated paper with the solvent) after 24 hours. The toxic effect of each product was expressed as the proportion of dead mosquitoes.

### Data Analysis

We used the same method to analyse the proportion of dead mosquitoes in toxicity assays and the proportion of escaped mosquitoes in both spatial repellency and contact irritancy assays. Data analysis was carried out using the R 2.12.2 software [24]. The proportions of escaped or dead mosquitoes in control and treated assays were compared using Fisher’s exact test. To take into account multiple testing, *P*-values of those tests were corrected according to Bonferroni using the Holm’s sequential method [25]. Generalized linear models (GLM) were fitted to assess the effects of products and concentrations on the proportions of escaped or dead mosquitoes using a binomial distribution with a logit-link function. To assess the adequacy of the models, residuals were checked graphically using a normal quantile-quantile plot. GLM coefficients relative to the effect “concentration × product” were compared to 0 and their significance tested using multiple comparison procedures for GLMs [26].

As previously described by Achee *et al.* [6], the proportions of escaped or dead mosquitoes were corrected by the control assay values using Abbot’s formula [27]. For all products and concentrations, these corrected proportions were used to perform a principal component analysis (PCA). Then, a hierarchical ascendant classification (HAC) based on Ward's algorithm was used to group the plant extracts based on the similarity of their effects using PCA-axes coordinates. This process yielded a binary segmentation tree, reflecting the hierarchy of similarities between responses to plant extracts. The optimal number of classes in the tree was determined by the decrease of the interclass variance (branch height-Appendix 1).

## Results

### Spatial Repellency Assays

**Figure 3:**
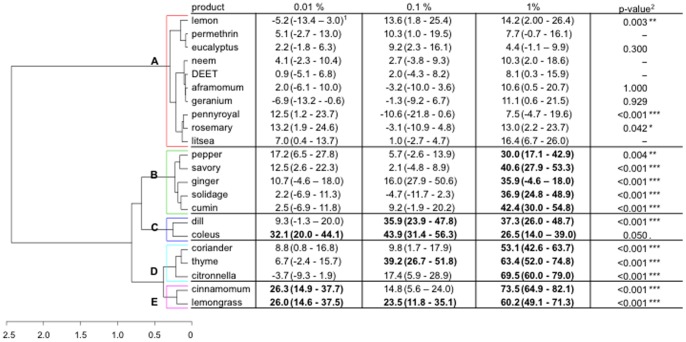
Response of four- to seven-day-old, non-blood-fed, sugar-fed, Kisumu strain of *Anopheles gambiae* females to the repellent effect of DEET, permethrin and 20 plant extracts at 3 concentrations (0.01, 0.1 and 1% of product in the solution on chromatographic papers): dendrogram determined by hierarchical ascendant classification and corrected proportion escaping using Abbott’s formula (confidence interval calculated with the Wald method) by treatment concentration. 1) Pairwise comparison of proportion was done using Fisher’s test. Values in bold lettering were significantly different from the control with the Holm’s sequential Bonferroni correction method. 2) P-value of the generalized linear model of the interaction concentration-product (dose-dependency) on the mosquito repellency. The coefficient was compared to zero so only the P-value of positive coefficient is given.

The spatial repellent effects of the different extracts significantly differed among plants (GLM, *P*<0.001) and were positively (model estimate: 0.82) associated with high concentrations of plant extracts (GLM, *P*<0.001) (Figure 3). Eight plant extracts did not exhibit a significant repellent effect at any concentration. These were lemon, eucalyptus, neem, aframomum, geranium, pennyroyal, rosemary, and litsea. Twelve out of the 20 plant extracts were found to be repellent at least at one concentration. These were pepper, savory, ginger, solidage, cumin, dill, coleus, coriander, thyme, citronella, cinnamon and lemongrass. Essential oils of lemongrass and coleus had a significant repellent effect at all concentrations tested. The two synthetic chemicals, DEET and permethrin were not repellent at 1% and below.

According to the similarity of the behavioural response, the clustering procedure based on HAC yielded 5 contrasted response classes. Class A grouped products that were not repellent, irrespective of their concentration. Classes B, C, D, and E grouped products that were significantly repellent at least at one concentration. Class B grouped 5 products that were efficient at only 1%. It is noteworthy that their activities slightly increased as concentration increased. Class C included two products that were repellent at several concentrations. For instance, coleus was repellent at all concentrations. These two products appeared to have a maximum efficiency of around 40%. Class D contained products that were repellent at least at one dose. The three products might be repellent at higher concentrations in agreement with their positive coefficients relative to the effect concentration. Class E regrouped the most repellent products for which a response was observed at least at two concentrations. Among the 20 plant extracts, the essential oils of lemongrass and cinnamon were the most repellent.

### Contact Irritancy Assays

**Figure 4:**
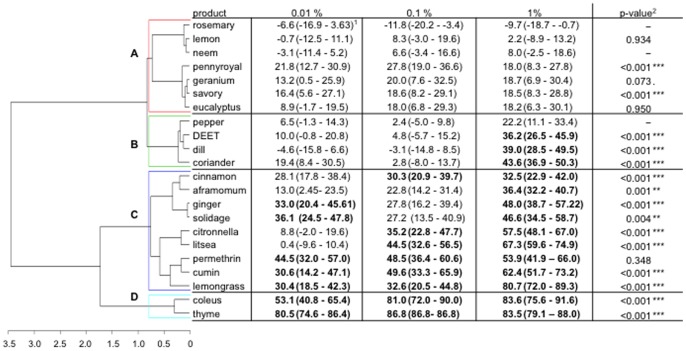
Response of four- to seven-day-old, non-blood-fed, sugar-fed, Kisumu strain *Anopheles gambiae* females to the irritant effect of DEET, permethrin and 20 plant extracts at 3 concentrations (0.01, 0.1 and 1% of product in the solution on chromatographic papers): dendrogram determined by hierarchical ascendant classification and corrected proportion escaping using Abbott’s formula (confidence interval calculated with the Wald method) by treatment concentration. 1) Pairwise comparison of proportion was done using Fisher’s test. Values in bold lettering were significantly different from the control with the Holm’s sequential Bonferroni correction method. 2) P-value of the generalized linear model of the interaction concentration-product (dose-dependency) on the mosquito irritancy. The coefficient was compared to zero so only the p-value of positive coefficient is given.

As observed in the repellency assays, the contact irritant activity of the 20 extracts significantly differed among plants (GLM, *P*<0.001) and increased with respect to the concentration of product (GLM, *P*<0.001, model estimate: 2.87) (Figure 4). Eight plant extracts had no irritant effects: rosemary, lemon, neem, pennyroyal, geranium, savory, eucalyptus and pepper. The other plant extracts, dill, coriander, cinnamon, aframomum, ginger, solidage, citronella, litsea, cumin, lemongrass, coleus and thyme, had irritant effects even at low concentrations. Similar to permethrin, cumin, lemongrass, coleus and thyme appeared irritant at all concentrations. Conversely, DEET was observed to be irritant at only 1%.

The HAC could be summarized by four response classes: Class A (8 products) containing products that were not irritant; Class B (4 products) that included products that were irritant at 1% concentration (except pepper oil), and whose interactions ‘products×concentration’ were significant, suggesting possible irritancy effects at higher concentrations; Class C (9 regrouped products) that were observed irritant at 2 or 3 concentrations included permethrin, which appeared to have a maximum escape threshold of around 50%; and class D (2 products) that were irritant at three concentrations and whose coefficients relative to ‘product×concentration’ interaction suggest that they might be irritant at lower concentrations. Among all plant extracts, coleus and thyme were the most irritant.

### Toxicity Assays

**Figure 5:**
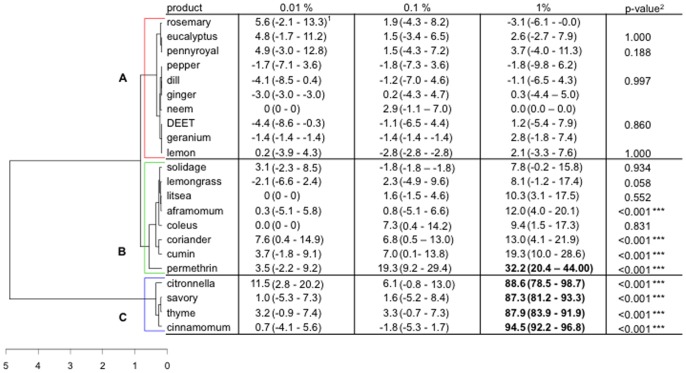
Responses of four- to seven-day old, non-blood-fed, sugar-fed, Kisumu strain of *Anopheles gambiae* females to the toxic effect of DEET, permethrin and 20 plant extracts at 3 concentrations (0.01, 0.1 and 1% of product in the solution on chromatographic papers): dendrogram determined by hierarchical ascendant classification and corrected mortality proportion using Abbott’s formula (confidence interval calculated with the Wald method) by treatment concentration. 1) Pairwise comparison of proportion was done using Fisher’s test. Values in bold lettering were significantly different from the control with the Holm’s sequential Bonferroni correction method. 2) P-value of the generalized linear model of the interaction concentration-product (dose-dependency) on the mosquito mortality. The coefficient was compared to zero so only the p-value of positive coefficient is given.

Plant extracts had varied toxicity, notably at the highest concentration tested (Figure 5). Once again, mortality rates were significantly influenced by both product and concentration (GLM, *P*<0.001 in both cases). The toxic activity was, therefore, positively influenced by increase in concentration (model estimate: 1.29). Sixteen plant extracts had no toxic effect, even at the highest concentration. These were rosemary, eucalyptus, pennyroyal, pepper, dill, ginger, neem, geranium, lemon, solidage, lemongrass, litsea, aframomum, coleus, coriander and cumin. In contrast, four plant extracts exhibited a toxic effect at 1%. These were cinnamon, citronella, savory and thyme. As expected, permethrin showed a toxic effect at 1%. Conversely, whatever the concentration, DEET did not appear efficient in killing mosquitoes. Knockdown response was not observed using either the plant extracts or the synthetic compounds.

The HAC analysis yielded three response classes: Class A (10 products) containing all products that were not toxic at all to mosquitoes, even at highest concentrations; Class B (8 products) that contained products with unclear effects at all concentrations (with one exception, permethrin) although toxicity slightly increased for aframomum, coriander and cumin, suggesting a potential toxicity at higher concentrations; and Class C (4 products) that appeared to be very toxic at 1%. Among the 20 plant extracts, cinnamomum, citronella, savory and thyme were the most toxic.

## Discussion

Our results showed that nearly all the 20 plant extracts tested had a significant effect on adults of the malaria vector *An. gambiae* (Table 2). Several were irritant or repellent but only a minority were toxic. For each of these three types of effects, several strong candidates were found. Some of these compounds presented interesting properties in more than one type of effect. These were cinnamon, citronella and thyme, which were shown to be repellent, irritant and toxic at the same time (Table 2). Compounds such as lemongrass, coleus, cumin and savory exhibited clear but restricted effects. Thyme is already known to have a toxic effect on Bruchidae [28], therefore, its mode of action might not be very specific. Rattan [29] showed that thymol, a natural monoterpene phenol found in oil of thyme, acts on the GABA system, reducing the neural inhibition, leading to hyper-excitation of the central nervous system, convulsions, and death. Thymol can also block the octopamine receptors that play a key role in the nervous transmission [29]. This certainly explains the irritant and toxic effects of thyme oil in our experiment.

**Table 2:** Synthesis of the behavioural response of *An. gambiae* females to DEET, permethrin and 20 plant extracts at 3 concentrations (0.01, 0.1 and 1% of product in the solution on chromatographic papers).

Common name	Scientific name	Repellenteffect	Irritanteffect	Toxic effect	Extract form
**DEET**		0	+	0	Synthetic compound
**Permethrin**		0	+++	+	Synthetic compound
**Aframomum**	*Aframomum pruinosum*	0	+	0	Essential oil
**Cinnamon**	*Cinnamomum zeylanicum*	++	++	+	Essential oil
**Citronella**	*Cymbopogon winterianus*	+	++	+	Essential oil
**Coleus**	*Plectranthus tenuicaulis*	+++	+++	0	Essential oil
**Coriander**	*Coriandrum sativum*	+	+	0	Essential oil
**Cumin**	*Cuminum cyminum*	+	+++	0	Essential oil
**Dill**	*Anethum graveolens*	++	+	0	Essential oil
**Eucalyptus**	*Eucalyptus globulus*	0	0	0	Essential oil
**Geranium**	*Pelargonium graveolens*	0	0	0	Essential oil
**Ginger**	*Zingiber officinalis*	+	++	0	Essential oil
**Lemon**	*Citrus limon*	0	0	0	Essential oil
**Lemongrass**	*Cymbopogon citratus*	+++	+++	0	Essential oil
**Litsea**	*Litsea cubeba*	+	++	0	Essential oil
**Neem**	*Melia azadirachta*	0	0	0	Vegetal oil
**Pennyroyal**	*Mentha pulegium*	0	0	0	Essential oil
**Pepper**	*Piper nigrum*	+	0	0	Essential oil
**Rosemary**	*Rosmarinus officinalis*	0	0	0	Biologic hydrolat
**Savory**	*Satureja montana*	0	0	+	Essential oil
**Solidage**	*Solidago canadensis*	+	++	0	Essential oil
**Thyme**	*Thymus vulgaris*	++	+++	+	Essential oil

0 = significant difference from the control with Fisher’s test,+ = significant difference from the control with Fisher’s test at one concentration.

Our results suggest that plant extracts exhibit different combinations of effects (i.e., spatial repellency, contact irritancy and/or toxicity). The magnitude of these effects differs among plant extracts and concentrations. For instance, irritancy, repellency and toxicity are, respectively, the primary, secondary and tertiary actions of thyme oil since these effects occur at low, medium and high concentrations, respectively. This contrasts with other plant extracts. The primary and secondary actions of dill oil are repellency and irritancy. This oil is not toxic on *An. gambiae* even at the highest concentration. This pattern suggests that the three effects observed here, i.e. repellency, irritancy and toxicity, involve different physiological mechanisms. Dekker *et al.* [30] showed that several repellent compounds elicit consistent electrophysiological responses in antennae of *Ae. aegypti.* The irritant effect of a product might be due to its action through tarsi on the nervous system [10]. Some individual compounds of essential oils are clearly detected and avoided by mosquitoes through their antennae. Still, the physiological influence of essential oils leading to repellency remains largely unknown [30], [31]. Deciphering the mechanisms underlying repellency might be challenging since this effect may be due to a synergistic effect of several compounds contained in plant extracts. Knowing the relation between the mechanism and behaviour could be of use in finding synergistic combinations. If our hypothesis is correct, (i.e. that irritancy, repellency and toxicity have independent modes of action), there may well be no cross-resistance, i.e. the resistance to one mode of action might not confer resistance to the other two modes of action. The evaluation of the relation between the mode of action and behaviour could be useful in reducing the risk of selecting resistant individuals. For example, linalool (the major compound of *C. sativum* essential oil), which showed a toxic effect on mosquitoes, was identified as an inhibitor of acetylcholinesterase [28]. Unfortunately, the efficacy of linalool on *An. gambiae* should be limited because resistance alleles at the acetylcholinesterase gene have already selected in West African populations of this species [32], [33], [34], [35]. The physiological mechanisms of plant extracts are largely unknown and interactions between individual compounds could be antagonistic, additive or synergistic. Since multiple resistance mechanisms could be involved, hypotheses on resistance development to essential oils are still speculative and need further investigations. Although neem oil has also been demonstrated to inhibit feeding behaviour [18], [36], [37], [38], it was not repellent because it is not volatile. Rosemary extract did not show any effect because hydrolats contain few active ingredients.

*An. gambiae* females were not significantly repelled or killed by DEET. This product showed only a contact irritancy effect at 1% (55 nmol/cm^2^). The vapour tension of DEET is low (0.27 Pa at 25°C) compared to other repellents such as p-menthane 3,8 diol (4.5 Pa at 25°C). Moreover, in our experiment, DEET was applied on a paper at 25°C rather than directly on skin (skin temperature is usually around 33°C), a difference that could explain the absence of repellent effect in the present investigation. Ditzen *et al.* [39] showed that DEET hides host odours (particularly 1-octen-3-ol) by inhibiting subsets of insect odorant receptors that require the OR83b co-receptor (masking effect). These olfactory receptor neurones (ORN) are involved in detecting semiochemicals that induce and facilitate host-seeking behaviour in mosquitoes [40]. However, according to Syed & Leal [41] ORN mosquitoes can detect and avoid DEET. In a sugar-feeding and surface-landing choice bioassay, mosquitoes did not land on DEET-treated paper and instead chose to land on solvent-treated paper. As a consequence, a ‘repellent’ may have more than one mode of action. DEET is reported as an inhibitor of acetylcholinesterase activity [42] and it was toxic on other species of mosquito at higher concentration [43] or with a different test method [44]. In our study, DEET did not showed toxic effect, that may be explained by a low concentration test or a product not enough bio-available. Our results showed that DEET is irritant but not repellent at a concentration equal to or below 1%. Indeed, we showed that without attractant bait and possible contact, adult mosquitoes did not avoid the tube containing DEET. According to Pickett *et al.* [45] a true behavioural repellent is a substance causing, at a distance, oriented movements away from the odour source. Thus, at 1% and 25°C, DEET cannot definitely be considered as a spatial repellent product.

Permethrin showed a contact irritant effect at 0.34 nmol/cm^2^, toxic and irritant effects at 3.4 nmol/cm^2^ and no spatial repellent effect. This corroborates the results of Achee *et al.* [6] on *Ae. aegypti.* In their experiments, permethrin was irritant and toxic at 2.5 nmoles/cm^2^ but did not appear repellent. Similarly, Dusfour *et al.* [46] showed that permethrin was irritant at 25 nmol/cm^2^ on *An. albimanus* but had no repellent effect. Pyrethroids are toxic because they modify the gating kinetics of the voltage-dependent sodium channel [47]. Their irritant effect might also be due to their influence on the nervous system. The low vapour pressure of permethrin (7×10^6^ Pa at 25°C) probably explains its lack of spatial repellency. Although pyrethroids are considered to have repellent, irritant and toxic effects [7], the treated bednets recommended by WHO could only be irritant and toxic [48] and not spatially repellent. In our study, the repellency of permethrin was not exhibited. However in field experiments the number of mosquitoes entering huts protected by a treated net was usually low compared to a non-treated net [49], indicating repellency. Finally, we must keep in mind that permethrin is a synthetic analogue of natural pyrethrins extracted from *C. cinerariaefolium*. Hence, we can consider that plants have already provided us good tools for managing mosquitoes [7].

Pyrethroids are widely used to control *An. gambiae* [2]. They are employed in bednet treatment, impregnation of cloths, indoor residual spraying and spatial treatments. The advantage of pyrethroids is their effectiveness at low dosages. They are also toxic, irritant, fast acting, stable and safe for humans [50]. Prioritization of toxic actions over spatial repellent and contact irritant actions should be balanced with the higher risk of rapid selection for resistance to the active compounds [6]. Additionally, the huge number of crop fields treated with pyrethroids indirectly speeds up the selection of resistant *An. gambiae* populations [50], [51], [52]. Pyrethroid resistance in *An. gambiae* might be due either to a mutation in the sodium channel sequence or to a higher metabolic detoxification through increase of enzyme activities [33]. Pyrethroids, like some of the plant extracts that were tested in the present study, are also irritant and toxic. Moreover, many plant extracts could have an effect on both host-seeking and -feeding behaviour [26]. Unfortunately, the knowledge gaps on repellents’ mode of physiological action has made it difficult to target the search for natural compounds to replace or synergize the DEET or the pyrethroids’ action [29]. Can some plant extracts be used as alternatives to pyrethroids and DEET? From our results, the most promising plant extracts are those from *C. winterianus, C. zeylanicum* and *T. vulgaris* because they combine the three effects. A mixture containing complementary active compounds and modes of action could reduce the selective pressure for resistance [53]. Plant extracts can be good candidates to find efficient spatial repellent, contact irritant or toxic products. They have been largely studied but their use is limited because of their volatility. Plant extracts evaporate quickly causing a rapid decline in efficacy. Fortunately, new technologies (e.g. gelatin–gum arabic microcapsules) can preserve a repellent effect for up to 30 days on treated fabric stored at room temperature (22°C) [54]. The mere addition of vanillin increases the efficacy duration of an essential oil [55]. Tawatsin *et al*. [55] showed that lemongrass oil with 5% vanillin had a repellent activity of 8 hours. Some commercialized products based on cinnamon oil, are already sold as insecticides and miticides [18]. It would be interesting to test such products against disease vectors like *An. gambiae.* Currently, it is difficult to impregnate a bednet with an essential oil that is both long-lasting and provides resistance to 20 washings as recommended by WHO [56]. Thus, the identification of compounds contained in active essential oils is a necessary step before carrying out specific technologies for material impregnation (L2I company, France, personal communication).

Consistent with their properties, essential oils might be useful for vector control. Their use will depend on their effects. The toxic effect could be useful in indoor residual spraying (IRS) or spatial spray treatment. Their irritancy effect could be suitable in IRS or treated bednet use. As indicated by the new WHO guidelines [57], the spatial repellency effect could also be a useful tool in vector control, as well as potential use as insect repellent (after safety tests), and in treatment of clothes or bednets. IRS, spatial spray, and repellent diffusers could also be considered. For instance, impregnating bednets with an irritant and repellent compound originating from essential oils for a long-lasting efficacy would be an interesting possibility. In addition, it would be particularly interesting, economically speaking, to choose essential oils from plants that are locally cultivated or with a rapid turnover in the wild. Amer & Mehlhorn [13] showed that cinnamom, citronella and lemongrass oils are repellent for three species of mosquitoes - *Ae. aegypti, Culex quinquefasciatus, An. stephensi*. The proportion of bites on arms treated with these essential oils was very close to zero. These authors also demonstrated an additive effect when using a blend of several essential oils extracted from *Litsea cubeba, Melaleuca leucadendron, M. quinquenervia, Viola odorata,* and *Nepeta cataria*. It is likely that a mixture of these five essential oils could be a suitable option in terms of personal protection because they do not have the same effects as some are irritant, others are repellent and a few might be toxic against these mosquitoes. However, these results cannot be extended to other mosquito species because variation in vector host-feeding preferences is known to trigger differential responses to essential oils [58].

The efficacy of the major compounds from the promising plant extracts (*C. winterianus, C. zeylanicum* and *T. vulgaris*) should be investigated to identified precisely their main mode of action and to determine if their combination have synergistic effects on *An. gambiae* and could be envisaged as serious alternatives to chemical insecticides for vector control.
